# Dynamic evolution analysis of the factors driving the growth of energy-related CO_2_ emissions in China: An input-output analysis

**DOI:** 10.1371/journal.pone.0243557

**Published:** 2020-12-16

**Authors:** Yan Ma, Zhe Song, Shuangqi Li, Tangyang Jiang

**Affiliations:** 1 Post-doctoral Research Station of Applied Economics, Zhongnan University of Economics and Law, Wuhan, China; 2 School of Business Administration, Zhongnan University of Economics and Law, Wuhan, China; 3 School of Economics and Business Administration, Chongqing University, Chongqing, China; 4 School of Accounting, Chongqing Technology and Business University, Chongqing, China; 5 School of Internet, Anhui University, Anhui, China; Institute for Advanced Sustainability Studies, GERMANY

## Abstract

In recent years, the global greenhouse effect caused by excessive energy-related carbon emissions has attracted more and more attention. In this paper, we studied the dynamic evolution of factors driving China's energy-related CO_2_ emissions growth from 2007 to 2015 by using energy consumption method and input-output analysis and used the IO-SDA model to decompose the energy carbon emissions. Within the research interval, the results showed that (1) on the energy supply-side, the high carbon energy represented by raw coal was still the main factor to promote the growth of energy-related CO_2_ emissions. However, the optimization of energy consumption structure is conducive to reducing emissions. Specifically, the high carbon energy represented by raw coal exhibited a downward trend in promoting the increment of energy-related CO_2_ emissions, while the clean energy represented by natural gas showed an upward trend in promoting the increment of CO_2_ emissions. It is worth noting that there is still a lot of room for optimization of China’s energy consumption structure to reduce emissions. (2) On the energy demand-side, the final demand effect is the main driving force of the growth of carbon emissions from fossil energy. Among them, the secondary industry plays a major role in the final demand effect. The "high carbonization" of the final product reflects the characteristics of China's high energy input in the process of industrialization. At the same time, since the carbon emission efficiency of the tertiary industry and the primary industry is better than that of the secondary industry, actively optimizing the industrial structure is conducive to slowing down the growth of carbon emission brought by the demand effect. (3) The input structure effect is the main restraining factor for the growth of energy carbon emissions, while the energy intensity effect has a slight driving effect on the growth of energy carbon emissions. The results show that China's "extensive" economic growth model has been effectively reversed, but the optimization of fossil energy utilization efficiency is still not obvious, and there is still a large space to curb carbon emissions by improving fossil energy utilization efficiency in the future.

## Introduction

At present, global warming caused by excessive carbon dioxide emissions has become a significant factor threatening the sustainable development of society [1]. In 2019, the statistics of Global Carbon Program suggested that global carbon emissions increased by about 2% in 2018 compared with 2017. This is another increase and a record high in global carbon emissions following a small increase in carbon emissions in 2017 (up 1.4%). Among them, carbon emissions from China accounted for 27% of global emissions and increased by about 4.5%. Meanwhile, after several consecutive years of decline, carbon emissions from the United States have also rebounded, with carbon emissions accounting for 15% of global emissions and increasing by 2.5%. It could be concluded that controlling the excessive carbon emissions has become a good way to protect the ecological environment and mitigate the greenhouse effect [2].

To reduce CO_2_ emissions and protect the ecological environment, the Chinese government has actively cooperated with other countries and international environmental protection organizations [3]. In 2009, China’s government officially announced that the intensity of carbon emissions per unit GDP by 2020 would be 40% to 45%, which was lower than that in 2005, at the Copenhagen Climate Conference [4]. In 2014, the Chinese government made a commitment at the Asia-Pacific Economic Cooperation (APEC) meeting that CO_2_ emissions would peak around 2030 and strive for a peak earlier; carbon dioxide emissions per unit GDP would decrease by 60% - 65% compared with 2005; non-fossil energy accounted for about 20% in primary energy consumption [5]. The above international commitments of the Chinese government illustrated China's determination to reduce carbon emissions and the inevitable choice for low-carbon economic development in the future.

In terms of sources, CO_2_ emissions were mainly from energy consumption [6], cement production [7], and biomass decomposition [8]. However, the main source of carbon dioxide emissions in China was energy-related carbon emissions according to statistics [9, 10]. Therefore, the focus of controlling China's excessive carbon dioxide emissions naturally fell on controlling energy-related carbon emissions. Existing literature on energy carbon emissions can be divided into energy supply-side carbon emissions [11, 12] and energy demand-side carbon emissions [13, 14]. For the supply-side, energy carbon emissions mainly come from the energy system [2]. For the demand-side, energy carbon emissions mainly come from the industrial system [2]. Therefore, the control of China’s industrial and energy system carbon emissions has become a key area for China to control energy carbon emissions.

With regard to the calculation of energy-related carbon emissions and analysis of factors driving the growth of energy-related carbon emissions, there are many mature methods at home and abroad that have conducted relevant research, such as the energy consumption method [15], the input-output method [16], the DEA model [17], the SDA model [18], and LMDI model [19]. Most of them focused on DEA model, SDA model or LMDI model to analyze the impact of various internal factors such as energy structure, energy intensity, input structure, final demand and industrial scale on carbon emissions. However, there are few literatures that quantitatively analyze the effects of various energy sources in the supply side energy system and industries in the demand side industry system on the growth of energy carbon emissions. Even if few scholars have done similar research, the scope of the research object is not comprehensive. For example, Jiang et al. (2019) used the energy consumption method and the input-output method to analyze the carbon emission structure of China’s industries and energy systems from 2002 to 2015, but did not analyze the impact of various energy sources and industries on carbon emission growth [2]. Jiang et al. (2020) uses the energy consumption method and the input-output method to analyze the evolution of the driving factors for the growth of electric energy-related carbon emissions from the energy supply-side and the energy demand-side in 2007–2015, but did not analyze the corresponding situation of fossil energy [20]. At the same time, since the energy system and industrial system are more practical for the implementation of emission reductions, the analysis of the effects of various energy sources and industries on the growth of energy carbon emissions in the past, which will provide realistic guidance and direction for subsequent focused emission reductions. Based on the above analysis, this paper used the energy consumption method and the input-output method to quantitatively analyze the evolution of the driving factors for the growth of energy-related carbon emissions from the fossil energy supply-side in China's energy system and the fossil energy demand-side in China's industrial system from 2007 to 2015, and applied the SDA model to decompose the carbon emission growth. Through the input-output framework, this study unifies the supply-side energy system carbon emission and the demand-side carbon emission, and quantitatively analyzes the effects of the various energy sources and industries in the energy system on the growth of energy carbon emissions. The framework provides a theoretical basis and practical direction for the follow-up energy system and industrial system to focus on emission reduction.

The rest of this paper is organized as follows. Section 2 reviewed and commented literatures about energy-related carbon emissions from the perspectives of the energy supply-side and demand-side. Section 3 described the data and model. Section 4 was the results and analysis. Section 5 was the conclusions and policy recommendations.

### Literature

The extant literature mainly focuses on energy-related carbon emissions from the perspective of the energy supply-side [12, 21] or the energy demand-side [22, 23]. Carbon emission analysis on the energy supply-side emphasizes the impact of energy supply on carbon emission growth in the energy system, while carbon emission analysis on energy demand-side emphasizes the impact of energy supply on carbon emission growth in the industrial system. Therefore, we summarized literatures on energy-related carbon emissions from the perspectives of the energy supply-side and the energy demand-side, and comprehensively analyzed the driving factors of energy consumption and carbon dioxide emissions. These works provide theoretical and practical significance for accounting carbon emission reduction.

### From the perspective of the supply-side

Many scholars studied energy-related CO_2_ emissions from the perspective of the energy supply-side, which was mainly based on CO_2_ emissions from various energy sources in the energy system. The non-renewable energy consumption had a positive impact on environmental degradation, while renewable energy consumption had a negative impact on environmental degradation, which helped to reduce environmental pollution [24]. Naturally, in the context of environmentally friendly development, fossil energy consumption and CO_2_ emissions have become a research topic that scholars cannot ignore and urgently need to solve. For example, Xu et al. (2014) studied the factors that influence carbon emissions due to fossil energy consumption in China, and found that the main driving force of CO_2_ emissions was economic output effect, followed by population scale and energy structure effects. The energy intensity effect was a major inhibitory factor [9]. Long et al. (2015) analyzed the relationship between CO_2_ emissions and energy consumption. The results indicated that that coal consumption played a leading role in economic growth and carbon emissions; the GDP had a two-way relationship with carbon emissions, coal, natural gas and power consumption; it was imperative to change the structure of energy consumption [25]. Yu et al. (2018) proposed a new economic-carbon emission-costs (ECC) multi-objective optimization model to measure the peak of CO_2_ emissions. The results showed that optimizing the coal-dominated structure of energy consumption would effectively contribute toward ensuring that China's carbon emissions peak by 2030. Meanwhile, the study found that the volume and time of the CO_2_ emission peak are not sensitive to renewable electricity price, but are sensitive to both the minimum average annual growth rates of GDP and the average annual reduction rate of energy intensity [26]. Zhao and Luo (2018) predicted the energy consumption structure of China by estimating the long-term and short-term relationship between carbon emission intensity, economic growth and consumption of natural gas, crude oil and coal. The results demonstrated that in the long run, the consumption of natural gas, crude oil and coal had a positive elasticity to GDP and carbon emission intensity, which implied that China should consider reducing the negative impact of CO_2_ emission intensity on economic growth and adjusting the optimal energy consumption structure [27]. By analyzing the relationship between energy consumption, CO_2_ emissions and economic growth in 68 emerging and developed countries from North Africa and Middle East, Muhammad (2019) found the increase in energy consumption led to the growth of CO_2_ emissions [28]. Meanwhile, the renewable energy consumption and CO_2_ emissions have also gradually attracted wide attention of scholars. However, the conclusions of these scholars are not unanimous. Although some scholars believed that renewable energy consumption could improve the growth of CO_2_ emissions [29, 30], Pata (2018) found total renewable energy consumption play no role in CO_2_ emissions reduction [31]. Nguyen and Kakinaka (2019) studied the relationship between CO_2_ emissions and renewable energy consumption at different development stages, which indicated the difference between high-income countries and low-income countries [32]. For high-income countries, the relationship between the consumption of renewable energy and output or CO_2_ emissions was just the opposite; For low-income countries, the relationship between the consumption of renewable energy was negatively and positively correlated with output and CO_2_ emissions, respectively. Moreover, some scholars made a comparative analysis for the relationship between renewable or non-renewable energy consumption and CO_2_ emissions [33–35], but its conclusion is based on data at the level of total energy consumption.

By summarizing the above literatures about carbon emissions from the supply-side perspective, it could be concluded that most of the literatures focused on the impact of energy intensity, population size, energy structure, and renewable energy on carbon emissions. The analysis of carbon emissions was also concentrated on the types with a large consumption structure such as coal and oil. Few literatures analyzed the evolution of the contribution of 17 different energy sources to the growth of energy-related carbon emissions in the entire energy system, which will provide scientific basis and theoretical basis for China's energy system to focus on the optimization of energy structure and carbon emissions reduction.

### From the perspective of the demand-side

A number of studies focus on energy-related carbon emissions from the perspective of the energy demand-side [36, 37], which was mainly based on the perspective industrial system. Due to the large proportion of construction, electricity, cement and heavy industry in China's industrial structure, these industries had attracted more attention [38–40]. However, some scholars studied the carbon emissions from other industries. For example, Wang et al. (2015) analyzed CO_2_ emissions of transportation infrastructure industry, and estimated CO_2_ emissions from four expressway projects in southwestern regions. The results showed that more than 80% of the carbon emissions from expressway projects came from raw material production, while material transportation and on-site construction accounted for only 3% and 10% of the total carbon dioxide emissions, respectively [41]. Tang et al. (2017) proposed a factor decomposition model to study the CO_2_ emissions of tourism. Their results manifested that the expansion of tourism scale and the growth of tourism output were the main reasons for the growth of CO_2_ emissions of tourism, and the decrease of energy intensity was beneficial to the reduction of tourism emissions [42]. Furthermore, some scholars decomposed the CO_2_ emissions in different industries. For example, Lin and Lei (2015) evaluated CO_2_ emissions of China's food industries, and found that energy intensity and industry activities were main factors affecting the increase and decrease of CO_2_ emissions in China's food industry, and optimizing the scale of industry and improving energy efficiency could effectively reduce carbon emissions [43]. Yang and Lin (2016) analyzed CO_2_ emissions in China's power industries, and pointed out that power intensity and economic activities were the main driving factors to promote the growth of carbon dioxide emissions; energy efficiency optimization had a significant effect on the CO_2_ emission reduction. In addition, other scholars have studied carbon emissions in other non-high energy-consuming industries [38]. Feng et al. (2018) decomposed CO_2_ emissions from China's metal industry, and drew a conclusion that the industrial output effect and the energy intensity effect were major factors for CO_2_ emissions growth and CO_2_ emissions reduction, respectively [44]. Du et al. (2018) analyzed CO_2_ emissions in six high-energy intensive industries of China. Their results demonstrated that the energy intensity effect was the prominent contributor for CO_2_ emissions reduction, while the industrial structure effect and the energy structure effect made small contribution to CO2 emissions change [45]. Gao et al. (2018) studied CO_2_ emissions in China's pharmaceutical industry, and found the production scale effect and the investment intensity effect were responsible for CO_2_ emission growth, while the energy intensity effect and the R&D efficiency effect were decisive for CO_2_ emissions reduction [46].

By summarizing the above literatures about energy-related carbon emission from the demand-side perspective, it could be concluded that most of the literatures focused on the carbon emissions of a certain industry and the impact of factors such as industrial growth, scale expansion and economic activities on the growth of carbon emissions. Few literatures analyzed the evolution of the contribution share of energy demand of various industries in the entire China’s industrial system to the growth of energy-related carbon emissions. This study can accurately and objectively reflect the impact of energy demand of various industries in the industrial system on the growth of carbon emissions, which provides scientific basis and theoretical basis for the China's industrial system to focus on the optimization of industrial structure and carbon emissions reduction.

### The academic contribution of this paper

Compared with previous research literatures about energy-related carbon emissions and driving factors, the contribution points of this paper are mainly reflected in the following three aspects. First, the fossil energy supply-side in energy system and the demand-side in industrial system have been unified to make up for the shortcomings of conducting research on energy-related carbon emissions from a single perspective in the past, and analyzed the impact of four effects on the growth of energy carbon emissions. Second, from the perspective of the supply-side, the driving factors and evolution of each specific fossil energy supply on the growth of energy-related carbon emissions were analyzed. Third, from the perspective of the demand-side, the driving factors and evolution of energy demand from various industries on the growth of energy-related carbon emissions were analyzed. The above three contribution points in this paper provide theoretical basis and data analysis for more efficient energy carbon emission control from the energy supply-side in the energy system and the energy demand-side in the industrial system.

## Data and model

### Data

Because the core data used in this paper is China's input-output table, which is updated every five years and extended every two or three years. As of the time when this paper is finished, we can get the latest input-output table is China's input-output table for 2015 that published in the 2019 China Statistical Yearbook. Therefore, the deadline of related data from input-output table in this paper is 2015. Meanwhile, because the input-output table before 2007 is relatively old, the research period of this paper is 2007–2015. Based on this, according to the research objectives of this paper, the data selected in this paper included China's energy balance tables for 2007, 2010, 2012 and 2015, and China's input-output tables for 2007, 2010, 2012 and 2015.

### Method and calculation

Based on the above analysis in introduction, this paper adopts the energy consumption method and the input-output method to analyze the impact of the energy system on the supply-side and the industry system on the demand-side on the growth of energy-related carbon emissions. We refer to the analytical model [44], and make corresponding modifications according to the research needs of this paper.

First, we use the energy consumption method to measure the CO_2_ emissions generated by the energy system on the supply-side. Carbon emissions from energy system are measured by the following calculation formula.

M=E×W (1)

where *M* is the carbon emissions generated by all energy sources combustion; *E* is the amount of each energy combustion; *W* is the carbon dioxide emission coefficient.

It is noteworthy that the energy system on the supply-side is composed of many energy sources. Due to differences of energy sources in the CO_2_ emission coefficients, the above-mentioned formula of carbon emission from energy system needs to be revised. The formula for calculating the CO_2_ emissions from the energy *i* for combustion is as follows.

$$M_{i}=E_{i}\times W_{i}$$ (2)

where *M*_*i*_ is the CO_2_ emissions from energy *i* for combustion; *E*_*i*_ is the amount of energy *i* for combustion; *W*_*i*_ is the carbon emission coefficient of energy *i*.

By summing up the CO_2_ emissions from all energy sources, the total CO_2_ emissions from energy system combustion on the supply-side can be derived. The summation formula is as follows.

$$M=\sum_{i}M_{i}=\sum_{i}E_{i}\times W_{i}$$ (3)

From the above analysis, we can draw that one of the key points of using energy consumption method to calculate carbon emissions of energy system was how to determine the total amount of energy combustion in energy system. By analyzing the China Energy Statistics Yearbook from 2003 to 2016, we find that 17 types of energy sources are listed in 2008 and 2011, while 20 types of energy sources are listed in 2013 and 2016. We summarize 17 types of energy sources for facilitating the research. From the perspective of energy consumption, energy consumption is divided into heat supply, thermal power generation, final consumption and the industrial raw materials. Among them, heat supply, thermal power generation and the final consumption belong to energy resource consumption for combustion, while the industrial raw materials does not involve energy combustion, so it needs to be eliminated. The total consumption of the energy *i* for combustion can be calculated by the following formula.

$$E_{i}=E_{i}^{T}+E_{i}^{P}+E_{i}^{H}-E_{i}^{M}$$ (4)

where *E*_*i*_ is the energy *i* consumption for combustion; $E_{i}^{T}$, $E_{i}^{P}$, $E_{i}^{H}$, $E_{i}^{M}$ are the consumption of the energy *i* for final consumption, thermal power generation, heat supply and industrial raw materials, respectively.

At the same time, when using the energy consumption method to measure carbon emissions, we also need to measure the CO_2_ emission coefficient of each type of energy. In the paper, the CO_2_ emission coefficient of each energy is calculated by multiplying the CO_2_ emission coefficient of each type of energy per unit heat and the average low calorific value of each energy. The calculation formula is as follows.

$$W_{i}=R_{i}\times Q_{i}$$ (5)

where *W*_*i*_ is the CO_2_ emission coefficient of energy *i*; *R*_*i*_ is carbon emission coefficient of energy *i* per unit heat; *Q*_*i*_ is the average low calorific value of energy *i* for combustion.

We have measured the carbon emission of energy system on the supply-side. Then we measure the carbon emissions of industries on the demand-side. The CO_2_ emissions include two parts, namely, CO_2_ emissions from intermediate demand (enterprises) and CO_2_ emissions from final demand (residents and governments).

Next, we utilize the input-output analysis to calculate CO_2_ emissions of China’s industry on the demand-side. When using input-output analysis to measure CO_2_ emissions on the demand-side, we need to pay attention to energy capital deposits and loans and energy export which will lead to CO_2_ emissions measurement errors on the demand-side besides excluding some energy sources used as industrial raw materials. Energy capital deposits and loans are saved in kind, so they will not generate carbon emissions; Energy exports will generate carbon emissions, but the place where carbon emissions occur is off-site. Therefore, it cannot be counted as China's carbon emissions. Firstly, we estimated the total energy investment of each energy industry *j* for combustion in the input-output table without excluding these two factors. The calculation formula is as follows.

$$K_{j}=KI_{j}+KF_{j}$$ (6)

where *K*_*j*_ is the total energy investment of each energy industry *j* for energy combustion; *KI*_*j*_ is the intermediate demand investment of energy industry *j* for energy combustion; *KF*_*j*_ is the final demand investment of energy industry *j* for energy combustion. In theory, we need to eliminate the two factors (energy capital deposits and loans, energy export). When these two factors are excluded, the formula for calculating the total investment of each energy industry for energy combustion is as follows.

$$K_{j}=KI_{j}+KF_{j}-KC_{j}-KE_{j}$$ (7)

where *KC*_*j*_ is the energy capital deposits and loans of energy industry *j* for energy combustion; *KE*_*j*_ is the energy export of energy industry *j* for energy combustion. Then, based on the previously calculated CO_2_ emissions from the energy combustion produced by the 4 energy industries and the amount of input of 4 energy industries in the input-output table to 42 other sectors, we measured CO_2_ emissions per unit input in 4 energy industries. The calculation formula is as follows.

$$T_{j}=\sum_{i}^{n}M_{i}$$ (8)

$$e_{j}=\frac{T_{j}}{K_{j}}$$ (9)

where *e*_*j*_ is CO_2_ emissions coefficient of energy industry *j* for energy combustion; *T*_*j*_ is total CO_2_ emissions from energy industry *j* for energy combustion. The CO_2_ emission coefficient of each energy industry *j* multiplied by the investment of the energy industry to each sector gets the carbon emissions from the sector consuming energy sources that are produced by energy industry on the demand-side. Due to 4 energy industries in input-output tables, the CO_2_ emissions of each sector are the sum of CO_2_ emissions from the sector consuming energy sources that are produced by 4 energy industries. The calculation formula is as follows.

$$E^{k}=\sum_{j}E_{j}^{k}=\sum_{j}C_{j}^{k}\times e_{j}$$ (10)

where *E*^*k*^ is CO_2_ emissions from industry *k* on the demand-side; $E_{j}^{k}$ is carbon emissions from energy industry *k* for energy combustion provided by energy industry *j* on the demand-side; $C_{j}^{k}$ is energy investment of the industry *k* in energy industry *j* for energy combustion part. We can measure the CO_2_ emissions of energy system on the supply-side and CO_2_ emissions of industries on the demand-side based on all the above formulas.

Next, we analyze the growth of CO_2_ emissions on the demand-side and supply-side, and calculate the contribution share of various factors to the growth of CO_2_ emissions on the demand-side and supply-side, so as to provide direction suggestions on how to carry out specific emission reduction.

From the perspective of carbon emissions on the supply-side, the calculation formulas for the contribution share of each type of energy to the carbon emissions increment of energy system on the supply-side is as follows.

$$W_{i}^{(k-n)k}=\frac{M_{i}^{k}-M_{i}^{k-n}}{\sum_{k=1}^{17}M_{i}^{k}-\sum_{k=1}^{17}M_{i}^{k-n}}$$ (11)

where $W_{i}^{(k-n)k}$ is the structure proportion of carbon emission increment of energy *i* in whole energy system from the year of *k*−*n* to the year of *k*; $M_{i}^{k}$ is CO_2_ emissions from energy *i* in the year of *k*; $M_{i}^{k-n}$ is CO_2_ emissions from energy *i* in the year of *k*−*n*; $\sum_{i=1}^{17}M_{i}^{k}$ is total carbon emissions from all 17 types of energy sources in the year of *k*; $\sum_{i=1}^{17}M_{i}^{k-n}$ is total carbon emissions from all 17 types of energy sources in the year of *k*−*n*.

From the perspective of CO_2_ emissions on the demand-side, on the one hand, the calculation formulas for the contribution share of final demand (residents and governments) to the carbon emissions increment on the demand-side is as follows.

$$P^{(k-n)k}=\frac{L^{k}-L^{k-n}}{\sum_{k=1}^{17}M_{i}^{k}-\sum_{k=1}^{17}M_{i}^{k-n}}$$ (12)

where *P*^(*k*−*n*)*k*^ is the contribution share of final demand to the carbon emissions increment on the demand-side from the year of *k*−*n* to the year of *k*; *L*^*k*^ is carbon emissions from final demand in the year of *k*; *L*^*k*−*n*^ is carbon emissions from final demand in the year of *k*−*n*. On the other hand, the calculation formulas for the contribution share of intermediate demand (enterprises) to the carbon emissions increment on the demand-side is as follows.

$$S^{(k-n)k}=\frac{R^{k}-R^{k-n}}{\sum_{k=1}^{17}M_{i}^{k}-\sum_{k=1}^{17}M_{i}^{k-n}}$$ (13)

where *S*^(*k*−*n*)*k*^ is the contribution share of intermediate demand to the carbon emissions increment on the demand-side from the year of *k*−*n* to the year of *k*; *R*^*k*^ is carbon emissions from intermediate demand in the year of *k*; *R*^*k*−*n*^ is carbon emissions from intermediate demand in the year of *k*−*n*. Moreover, we analyzed the impact of various industries with energy demand on carbon emission increment in China. The formula for calculating the contribution of each sector to the growth of energy-related CO_2_ emission in China's industry is as follows.

$$P_{(i-n)i}^{k}=\frac{E_{i}^{k}-E_{i-n}^{k}}{\sum_{k=1}^{9}E_{i}^{k}-\sum_{k=1}^{9}E_{i-n}^{k}}$$ (14)

where $P_{(i-n)i}^{k}$ is the contribution share of sector *k* to the CO_2_ emissions increment on the demand-side from the year of *i*−*n* to the year of *i*; $E_{i}^{k}$ is carbon emissions from sector *k* the year of *i*; $E_{i-n}^{k}$ is carbon emissions from sector *k* in the year of *i*−*n*; $\sum_{k=1}^{9}E_{i}^{k}$ is the total carbon emissions from all 9 sectors in the year of *i*. $\sum_{k=1}^{9}E_{i-n}^{k}$ is carbon emissions from all 9 sectors in the year of *i*−*n*.

$$SE_{i}=\frac{W_{i}}{P_{i}}$$ (15)

Where *SE*_*i*_ is the carbon emission sensitivity coefficient of energy *i*; *W*_*i*_ is the standard coal conversion coefficient of energy *i*; *P*_*i*_ is the coefficient of carbon emission of energy *i*.

Next, we build an IO-SDA model to decompose the structural factors of energy carbon emission growth [47], which can be decomposed into four factors: final demand effect, input structure effect, energy intensity effect, and energy structure effect [48].

AX+Y=X (16)

$$\left(\begin{array}{c} X_{1}\\ X_{2}\\ \vdots \\ X_{i} \end{array}\right)=\left(\begin{array}{cccc} A_{11} & A_{12} & \cdots & A_{1j}\\ A_{21} & A_{22} & \cdots & A_{2j}\\ \vdots & \vdots & \ddots & \vdots \\ A_{i1} & A_{i2} & \cdots & A_{ij} \end{array}\right)\left(\begin{array}{c} X_{1}\\ X_{2}\\ \vdots \\ X_{i} \end{array}\right)+\left(\begin{array}{c} Y_{1}\\ Y_{2}\\ \vdots \\ Y_{i} \end{array}\right)$$ (17)

Where, *X* = [*X*_*j*_] is the total output matrix, *Y* = [*Y*_*j*_] is the final demand matrix, and *A* is the direct consumption matrix.

In the input-output model, fossil energy participates in national economic production as an intermediate input in the fossil energy sector. It can be concluded that China’s fossil energy carbon emissions *E*_*h*_ can be expressed as:

$$E_{h}=FSILY$$ (18)

$$\begin{array}{c} {\Delta E}_{h}=\underset{energy\;structure\;effect}{\underbrace{\frac{1}{2}\left(F_{t}I_{t}L_{t}Y_{t}-F_{t-1}I_{t-1}L_{t-1}Y_{t-1}\right)\Delta S}}\underset{energy\;intensity\;effect}{+\underbrace{\frac{1}{2}\left(F_{t}S_{t}L_{t}Y_{t}-F_{t-1}S_{t-1}L_{t-1}Y_{t-1}\right)\Delta I}}\\ \underset{input\;structure\;effect}{+\underbrace{\frac{1}{2}\left(F_{t}S_{t}I_{t}Y_{t}-F_{t-1}S_{t-1}I_{t-1}Y_{t-1}\right)\Delta L}}\underset{final\;demand\;effect}{+\underbrace{\frac{1}{2}\left(F_{t}S_{t}I_{t}L_{t}-F_{t-1}S_{t-1}I_{t-1}L_{t-1}\right)\Delta Y}} \end{array}$$ (19)

Where, *S* is the energy structure matrix, *F* is the carbon emission coefficient matrix of each fossil energy, *I* is the energy consumption intensity matrix, and *L* is the Leontief inverse matrix. Then the growth of fossil energy carbon dioxide emissions is Δ*E*_*h*_.

## Results and analysis

### Calculation of energy-related CO_2_ emissions from 2007 to 2015

**Fig 1:**
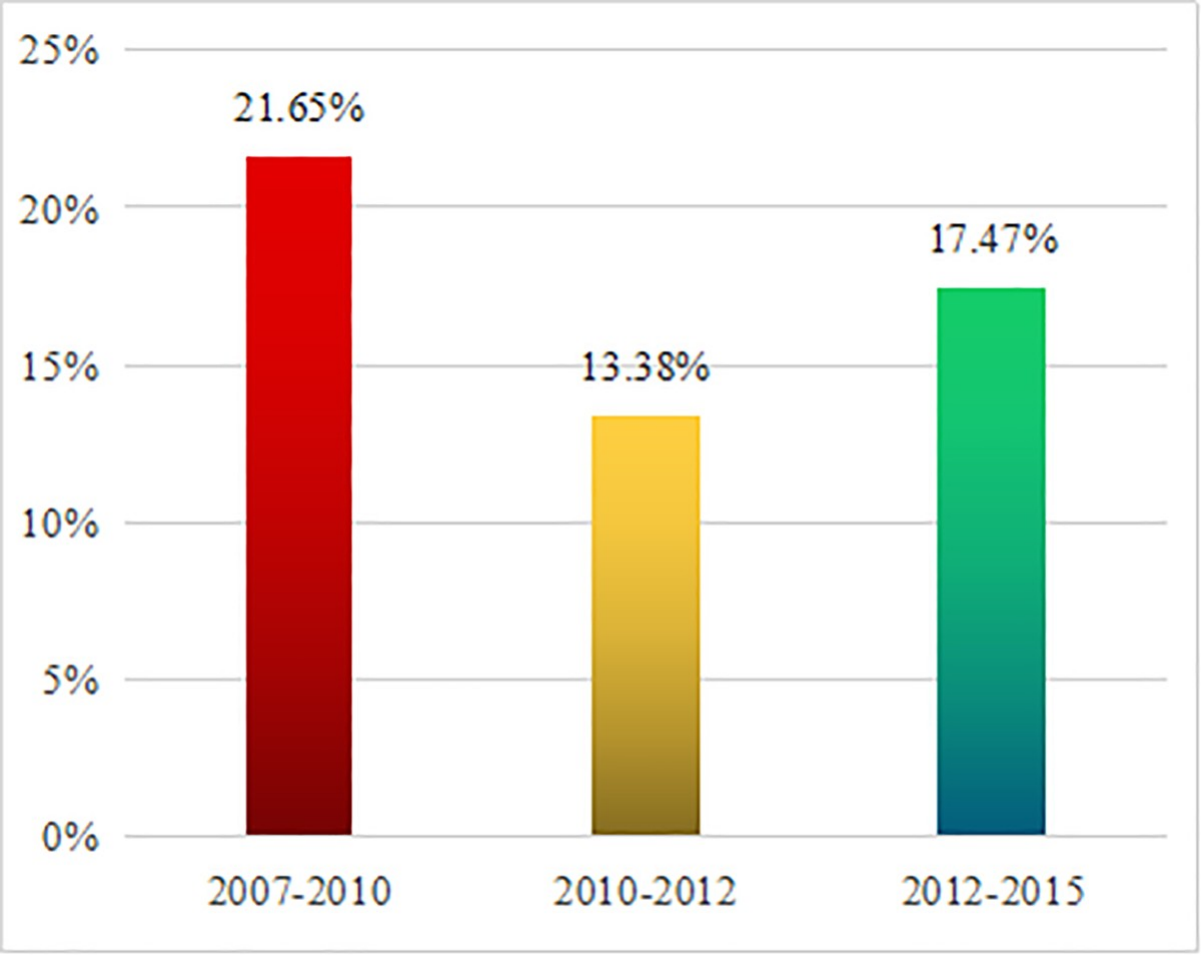
The growth rate of energy-related CO_2_ emissions.

We could calculate China's energy-related carbon emissions in 2007, 2010, 2012 and 2015 according to the above energy consumption method. The growth rate of energy-related CO_2_ emissions was shown in Fig 1.

According to the Fig 1, China's energy-related CO_2_ emissions increased by 1510.132 million tons in 2010, 1135.492 million tons in 2012, and 1681.177 million tons in 2015 compared with 2007, 2010, 2012, respectively. It could be concluded that the increase rate of energy-related CO_2_ emissions showed a downward trend from 2007 to 2015. Specially, the growth rate of energy-related CO_2_ emissions was 21.65% from 2007 to 2010, 13.38% from 2007 to 2012, and 17.47% from 2012 to 2015.

### Analysis of the contribution of various energy sources to the CO_2_ emissions growth of energy system on supply-side

**Fig 2:**
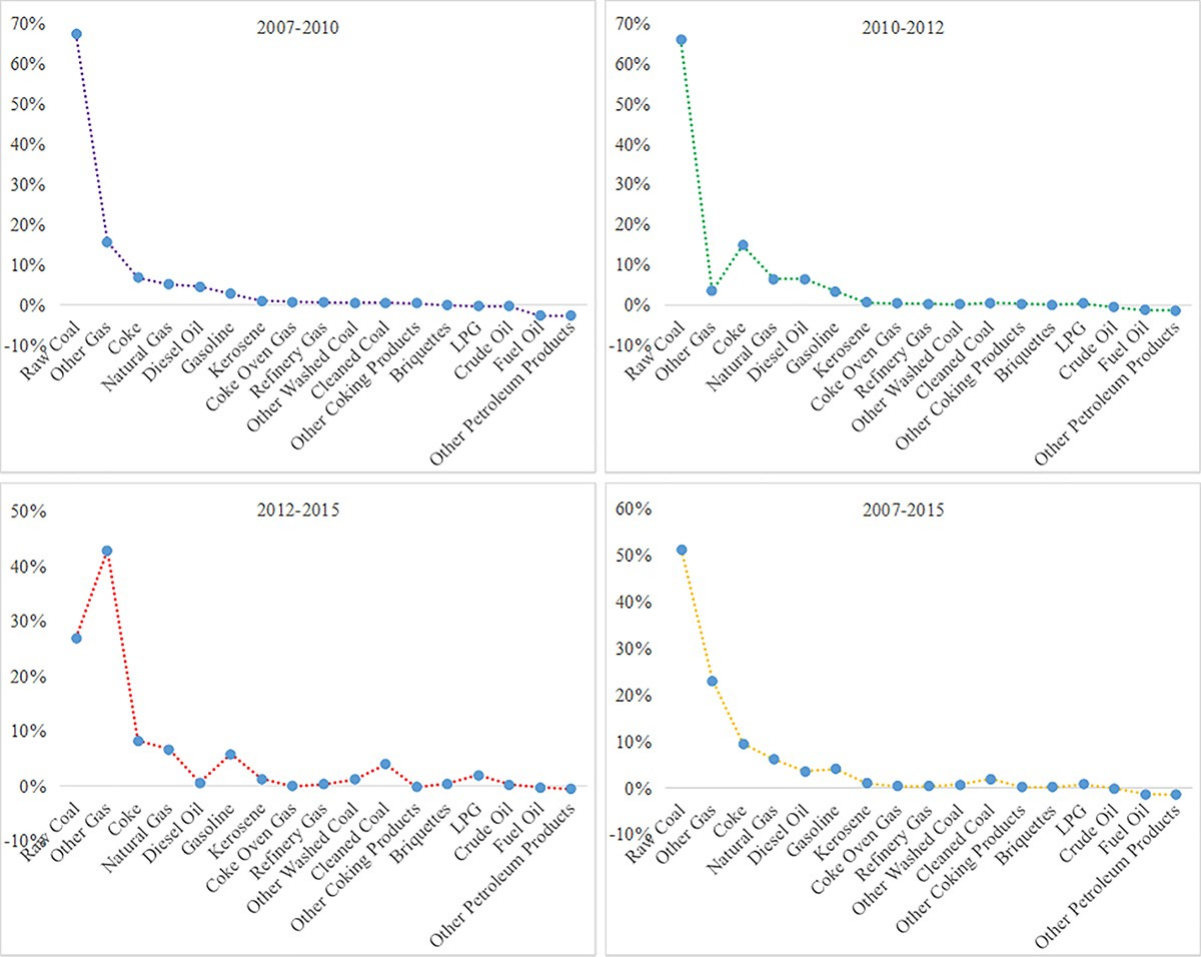
Contribution of various energy sources to CO_2_ emissions growth from 2007 to 2015.

**Fig 3:**
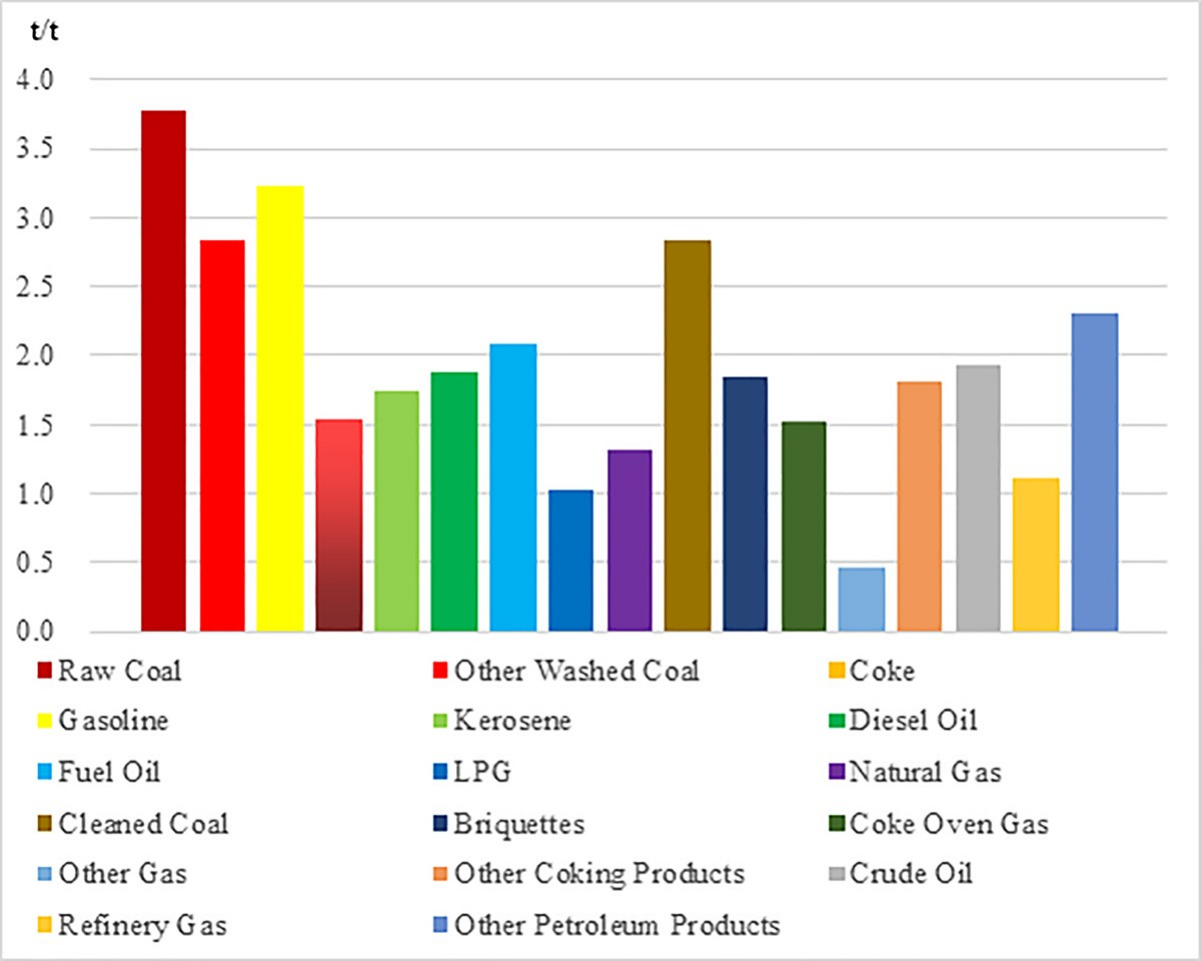
The carbon emission sensitivity of each energy consumption.

The share of each type of energy to the CO_2_ emissions increment in energy system was shown in Fig 2. The carbon emission sensitivity of each energy consumption was shown in Fig 3.

As shown in Fig 2, in the view of time evolution, the contribution share of high-carbon energy sources, such as other coking products, raw coal and diesel oil, to China's energy carbon emission growth showed a downward trend from 2007 to 2015. Specifically, contribution of raw coal to China's carbon emission growth was 67.35%, 65.86%, 26.94% during 3 sub-periods (2007–2010, 2010–2012, 2012–2015), respectively; other coking products contributed 0.45%, 0.32%, -0.19% to China's energy-related carbon emissions increment during above 3 sub-periods; diesel oil contributed 4.53%, 6.41%, 0.64% to China's energy carbon emission increment during the same 3 sub-periods. This change benefited from the Chinese government actively carrying out the optimization of energy consumption structure, controlling the excessive growth of high-carbon energy consumption proportion (especially the proportion of high-carbon energy such as coal and diesel oil), vigorously promoting technological progress, and improving energy efficiency. These initiatives led to the contribution share of raw coal, other coking products and diesel oil to China's energy-related carbon emissions growth showing a fluctuating downward trend.

In addition, the contribution share of other gas and natural gas, as clean energy sources, to energy carbon emission increment showed a rising trend. Concretely, natural gas contributed 5.10%, 6.46%, 6.68% to China's energy carbon emission increment during the same 3 sub-periods, and the other gas contributed 15.60%, 3.45%, 42.87% to China's energy-related carbon emissions increment. This was because the Chinese government not only controlled the rapid increment of high-carbon energies such as raw coal, but also actively promoted the diversification of energy sources, and deepened the processing of extensive energy sources, and focused on the development of clean low-carbon energies such as natural gas. As a result, the contribution of clean energy sources to energy-related carbon emissions was increasing.

From the above analysis, we concluded that, from 2007 to 2015, although the structure of China's energy consumption has been optimized, the high-carbon energy sources were still the main factor to promote the growth of CO_2_ emissions.

As shown, Fig 3 presented the carbon emission sensitivity of each energy consumption. We concluded that the carbon emission coefficients of each energy source are significantly different. Among them, raw coal, other washed coal, coke, cleaned coal and other energy sources have higher carbon emission sensitivity coefficient, which means that the carbon emission generated by burning these energy sources per unit standard coal is large. However, the carbon emission sensitivity coefficient of natural gas, other gas, liquefied petroleum gas and refinery dry gas is relatively low, which means that the carbon emission generated by burning these energy sources per unit standard coal is relatively small. Through the above analysis, it can be concluded that reducing the proportion of high carbon energy consumption structure, such as raw coal, other washed coal, coke and cleaned coal, while increasing the proportion of energy consumption structure, such as natural gas, other gas, liquefied petroleum gas and refinery dry gas, which is conducive to slowing down the growth of carbon emission caused by the growth of energy consumption.

### Analysis of the contribution of various industries to the CO_2_ emission growth of industrial system on demand-side

**Fig 4:**
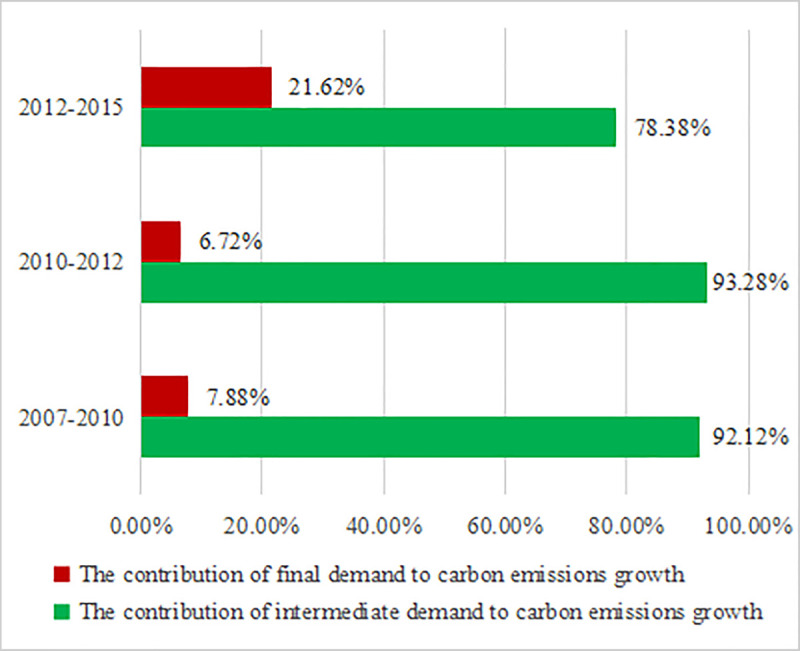
The contributions of intermediate demand and final demand to carbon emissions growth from 2007 to 2015.

The energy-related carbon emissions on the demand-side were not entirely accomplished by enterprises, but included two parts: intermediate demand (enterprises) and final demand (residents and governments). The contribution share of intermediate demand and final demand to the increment of total CO_2_ emissions was shown in Fig 4.

The analysis of Fig 4 led to the fact that the growth of energy-related CO_2_ emissions from 2007 to 2015 was still mainly driven by intermediate demand, while the final demand played a smaller role. In 2007–2010, the contribution share of intermediate demand to carbon emission growth was 92.12%, while the contribution share of final demand to carbon emission growth was 7.88%. In 2010–2012, the contribution share of intermediate demand to carbon emission growth was 93.28%, while the contribution share of final demand to carbon emission growth was 6.72%. In 2012–2015, the contribution share of intermediate demand to carbon emission growth was 78.38%, while final demand contributed 21.61% to the growth of carbon emissions. Therefore, it could be concluded that the contribution share of intermediate demand to carbon emissions growth exhibited a slow downward trend, while the contribution share of final demand to carbon emissions growth showed a slow upward trend.

**Fig 5:**
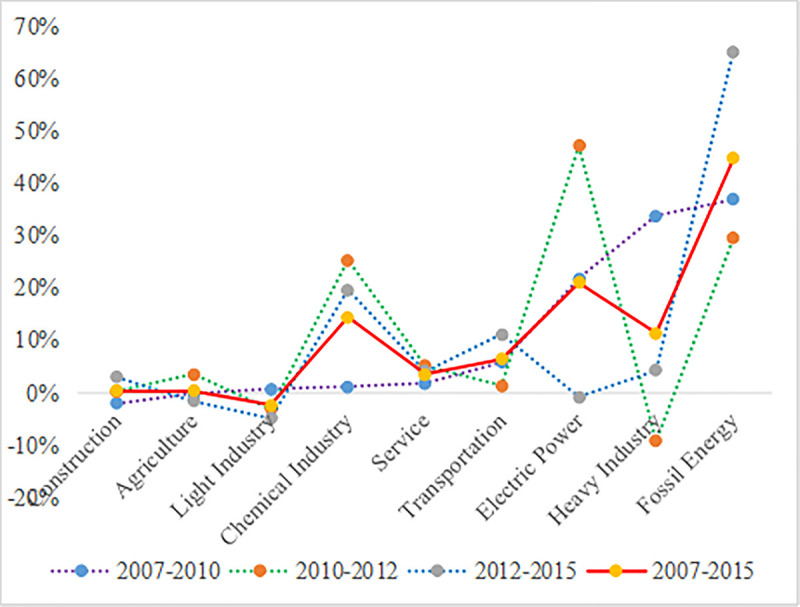
Contribution shares of various sectors to carbon emission growth from 2007 to 2015.

In China's input-output table, the industrial structure was divided into 42 sectors, but we merged them into 9 sectors [2]. The trend chart of contribution share of each industry to carbon emission growth along with time evolution was shown in Fig 5.

As shown in Fig 5, from the perspective of industrial structure, from 2007 to 2010, fossil energy, heavy industry, electric power, transportation and chemical industry contributed larger share to China's industrial carbon emission growth on the demand-side, while agriculture and construction had negative effects on China's industrial carbon emission growth on the demand-side. From 2010 to 2012, contribution share of the electric power, fossil energy, chemical industry and service sector to the growth of industrial CO_2_ emissions on the demand-side were larger, while contribution share of light industry and heavy industry to the growth of industrial CO_2_ emissions on the demand-side were negative. From 2012 to 2015, the electric power, agriculture and light industry had negative effects on the growth of industrial CO_2_ emissions on the demand-side, while the fossil energy, chemical industry, transportation and heavy industry contributed a larger share to the growth of industrial CO_2_ emissions on the demand-side.

According to the above analysis, we concluded that the main sectors to promote the growth of industrial CO_2_ emissions on the demand-side were fossil energy sector, electric power, heavy industry, chemical industry and transportation, while the service industry, construction and agriculture played a smaller role in promoting the growth of industrial CO_2_ emissions on the demand-side. At the same time, light industry had a negative effect on the increment of CO_2_ emissions.

**Fig 6:**
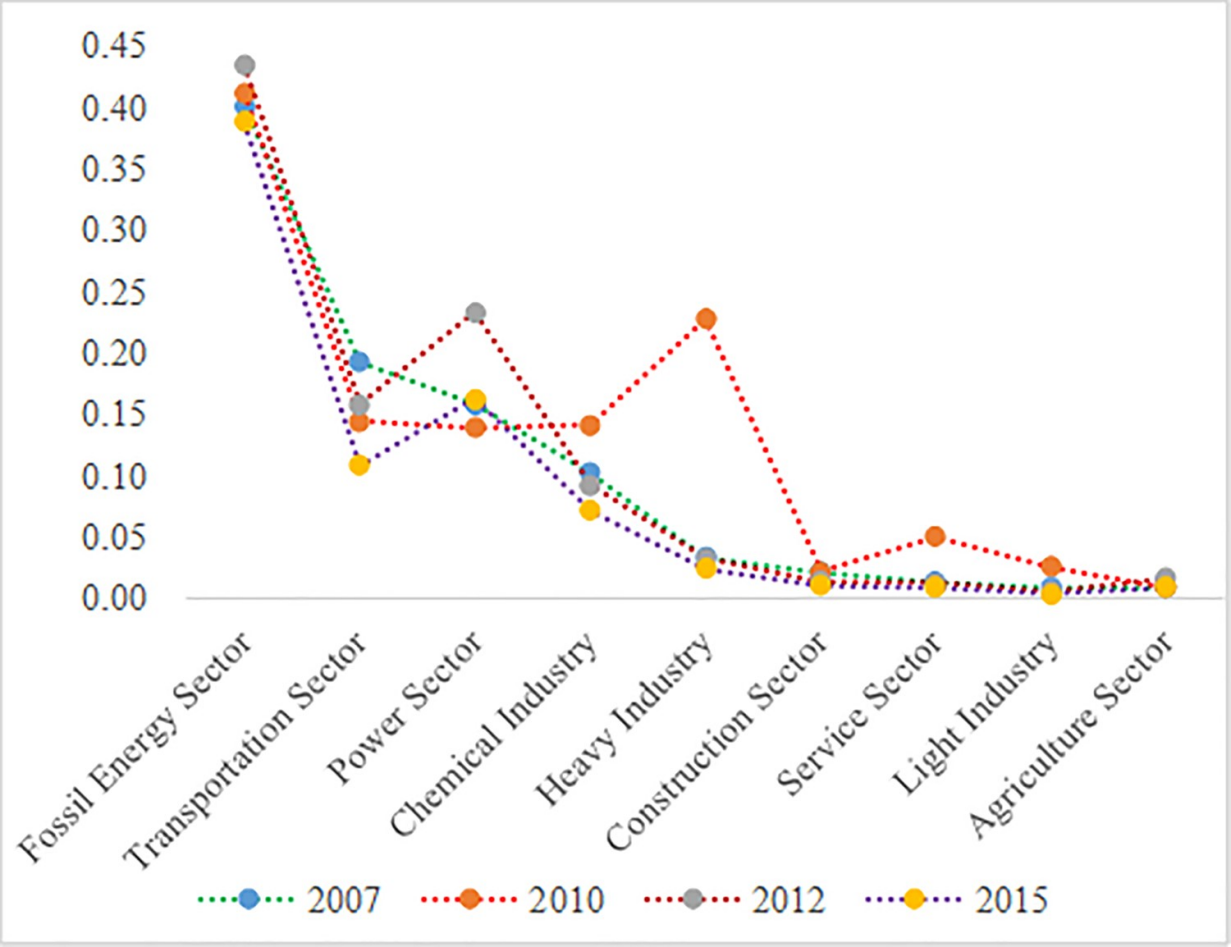
Direct consumption coefficient of fossil energy by various industries.

Next, we analyzed the impact of industrial structure changes on CO_2_ emission growth. The direct consumption coefficient of fossil energy by various industries were shown in Fig 6 (Ton/10,000 RMB).

According to the above Fig 6, we concluded that the direct consumption coefficient of fossil energy from the primary and tertiary industries was prior to that of the secondary industries (heavy industry, chemical industry, fossil energy sector, etc.), which indicated that expansion in the secondary industry on the same scale could produce more carbon emissions than that of the tertiary industry or the primary industry. Therefore, industrial structure optimization could make contribution to the carbon emissions reduction.

Table 1 shows the four-factor decomposition results obtained by using the I0-SDA model.

**Table 1:** Decomposition results of the four-factors from 2007 to 2015.

Effect of name	Energy structure effect	Energy intensity effect	Input structure effect	Final demand effect
Impacts of various effects on the growth of energy carbon Emissions (unit: TONS)	-145751.2	9300.03	-612899.63	1274340.97
The contribution share of each effect in the total effect	-27.04%	1.73%	-113.70%	236.41%

From the analysis of results in Table 1, it can be concluded that the positive and negative effects of the four-factors on the growth of China's energy carbon emissions from 2007 to 2015 and the intensity of the effects are significantly different. To be specific, first, the final demand effect is the main driving force of the growth of carbon emissions from fossil energy, which is consistent with the research results of Liao and Xu (2017). The "high carbonization" of the final product reflects China's high energy input in the process of industrialization. Second, energy intensity has a weak driving effect on the growth of carbon emissions from fossil fuels. The result indicates that the optimization of fossil energy utilization efficiency in China is still not obvious during the study period, and there is still a large space to curb carbon emissions by improving fossil energy utilization efficiency in the future. Third, the input structure effect is the main restraining factor for the growth of fossil energy carbon emissions. The results show that China’s extensive pattern of economic growth has been effectively reversed from 2007 to 2015. Fourth, the energy structure effect has an inhibitory effect on the growth of carbon emissions. This reflects that the national "Eleventh Five-Year Plan" and "Twelfth Five-Year Plan" proposed to reduce the proportion of raw coal consumption and increase the proportion of low-carbon consumption such as natural gas have been well implemented.

### Conclusions and policy recommendations

By using the energy consumption method and input-output method, we calculated and analyzed the contribution share of energy sources and industries to energy-related carbon emissions growth from 2007 to 2015 on the supply-side and demand-side. At the same time, we used the IO-SDA model to decompose the energy carbon emissions. Within the research interval, the research results show that (1) on the energy supply-side, the high carbon energy represented by raw coal was still the main factor to promote the growth of energy-related CO_2_ emissions. However, the optimization of energy consumption structure is conducive to reducing emissions. Specifically, the high carbon energy represented by raw coal exhibited a downward trend in promoting the increment of energy-related CO_2_ emissions, while the clean energy represented by natural gas showed an upward trend in promoting the increment of CO_2_ emissions. It is worth noting that there is still a lot of room for optimization of China’s energy consumption structure to reduce emissions. (2) On the energy demand-side, the final demand effect is the main driving force of the growth of carbon emissions from fossil energy. Among them, the secondary industry plays a major role in the final demand effect. The "high carbonization" of the final product reflects the characteristics of China's high energy input in the process of industrialization. At the same time, since the carbon emission efficiency of the tertiary industry and the primary industry is better than that of the secondary industry, actively optimizing the industrial structure is conducive to slowing down the growth of carbon emission brought by the demand effect. (3) The input structure effect is the main restraining factor for the growth of energy carbon emissions, while the energy intensity effect has a slight driving effect on the growth of energy carbon emissions. The results show that China's "extensive" economic growth model has been effectively reversed, but the optimization of fossil energy utilization efficiency is still not obvious, and there is still a large space to curb carbon emissions by improving fossil energy utilization efficiency in the future.

According to the conclusions, we put forward the following policy recommendations on CO_2_ emissions reduction. (1) The growth of CO_2_ emissions mainly came from the combustion of high-carbon energy represented by raw coal and washed coal on the supply-side; On the contrary, natural gas, as a clean energy, played a less promotive role. Therefore, it was suggested to further decrease (increase) the supply of high-carbon (low-carbon) energy so as to optimize the energy structure (2) The power sector, heavy industry, fossil energy sector, chemical industry and transportation industry belonged to the industry with larger carbon emissions, while agriculture, construction, service industry and light industry belonged to the industry with smaller carbon emissions. Naturally, the Chinese government should focus on emissions reduction in enterprises with large carbon emissions such as the power sector, heavy industry, fossil energy and other sectors. Especial for the fossil energy sector and the chemical industry, their growth of energy-related CO_2_ emissions had not decreased but had increased. Therefore, the follow-up emission reduction work needed to guide the two industries to increase the intensity of emission reduction through policies. (3) Because the efficiency of carbon emission from the primary and tertiary industries was prior to that of the secondary industries. It was necessary to further increase the structural proportion of services and agriculture sectors, and reduce the structural proportion of fossil energy sector and heavy industry in the secondary industry in industrial structure. (4) Since energy intensity effect and input structure effect are the main ways for China to reduce emissions in the long-term, it is the main entry point for future industrial emission reduction to actively upgrade and innovate energy use technologies and improve energy utilization efficiency.

## References

[1] Henson S. A., Beaulieu C., Ilyina T., John J. G., Long M., Séférian R., et al, 2017 Rapid emergence of climate change in environmental drivers of marine ecosystems. Nat. Commun. 8, 14682 10.1038/ncomms14682 28267144 PMC5343512

[2] Jiang T., Huang S., Yang J., 2019 Structural carbon emissions from industry and energy systems in China: An input-output analysis. J. Cleaner Prod. 240, 118116.

[3] Jiang X., Green C., 2017 China’s future emission reduction challenge and implications for global climate policy. Climate Policy 18, 889–901.

[4] Risinger C. F., 2009 Teaching about U.S. Climate Policy and the 2009 Copenhagen Conference. Social Education 73, 279–281.

[5] Xie Y., Zhao B., Zhao Y., Luo Q, Wang S, Bai S, 2017 Reduction in population exposure to PM 2.5, and cancer risk due to PM 2.5 -bound PAHs exposure in Beijing, China during the APEC meeting. Environ. Pollut. 225, 338–345. 10.1016/j.envpol.2017.02.059 28284555

[6] Jia J., Gong Z., Xie D., Chen J., Chen C., 2018 Analysis of drivers and policy implications of carbon dioxide emissions of industrial energy consumption in an underdeveloped city: The case of Nanchang, China. J. Cleaner Prod. 183, 843–857.

[7] Liu Z., Guan D., Wei W., Davis S. J., Ciais P., Bai J., et al, 2015 Reduced carbon emission estimates from fossil fuel combustion and cement production in China. Nature 524(7565), 335–338. 10.1038/nature14677 26289204

[8] Duan W., Gong J., Zhou M., Chen L., Zhang Y., Li M., et al, 2017 Effects of Gap Size and Dead Leaf Decomposition on Soil Microbial Biomass Carbon in Different Stand Types of Natural Pinus koraiensis Mixed Forest. Forest Research 30, 268–275.

[9] Xu S., He Z., Long R., 2014 Factors that influence carbon emissions due to energy consumption in China: Decomposition analysis using LMDI. Appl. Energy 127,182–193.

[10] Li J., Sun C., 2018 Towards a low carbon economy by removing fossil fuel subsidies? China Econ. Rev. 50, 17–33.

[11] Wang C., Dan Z., Tsutsumi A., You S., 2017 Sustainable energy technologies for energy saving and carbon emission reduction. Appl. Energy 194, 223–224.

[12] Li K., Chen C., Lai J., Wang Y., 2013 Abatement cost analysis in CO2 emission reduction costs regarding the supply-side policies for the Taiwan power sector. Energy Policy 61, 551–561.

[13] Zhang Y., Da Y., 2015 The decomposition of energy-related carbon emission and its decoupling with economic growth in China. Renew. Sust. Energ. Rev. 41, 1255–1266.

[14] Wang Z., Ye D., 2017 Forecasting Chinese carbon emissions from fossil energy consumption using non-linear grey multivariable models. J. Cleaner Prod. 142, 600–612.

[15] Sheinbaum C., Ruíz B. J., Ozawa L., 2011 Energy consumption and related CO2 emissions in five Latin American countries: Changes from 1990 to 2006 and perspectives. Energy 36, 3629–3638.

[16] Liu Y, Lv Y, Zhou M., 2015 Analysis on the Influence Factors of Computing CO2 Emission by Input-output Method. China Population, Resources and Environment 25, 21–28.

[17] Chen Z, Wanke P, Antunes J J M, Zhang N., 2017 Chinese airline efficiency under CO2 emissions and flight delays: A stochastic network DEA model. Energy Econ. 68, 89–108.

[18] Xu S, Zhang L., Liu Y., Zhang W., He Z., Long R., et al, 2017 Determination of the factors that influence increments in CO2 emissions in Jiangsu, China using the SDA method. J. Cleaner Prod. 142, 3061–3074.

[19] Moutinho V., Madaleno M., Inglesi-Lotz R., Dogan E., 2018 Factors affecting CO2 emissions in top countries on renewable energies: A LMDI decomposition application. Renew. Sust. Energ. Rev. 90, 605–622.

[20] Jiang T, Yang J, Huang S., 2020 Evolution and driving factors of CO2 emissions structure in China’s heating and power industries: The supply-side and demand-side dual perspectives. J. Cleaner Prod. 264: 121507.

[21] Shrestha R. M., Marpaung C. O. P., 2005 Supply- and demand-side effects of power sector planning with demand-side management options and SO emission constraints. Energy Policy 33, 815–825.

[22] Gao C., Li W., Pan W., Ma Y., 2009 Management model of carbon emission reduction in chemical industry based on the improvement of overall benefits. Modern Chemical Industry 81, 337–345.

[23] Shi Y., Chen L., Zhen L., Yan J., Hu J., 2012 Analysis on the Carbon Emission Reduction Potential in the Cement Industry in Terms of Technology Diffusion and Structural Adjustment: A Case Study of Chongqing. Energy Procedia 16, 121–130.

[24] Sharif A., Raza S. A., Ozturk I., et al, 2019 The Dynamic Relationship of Renewable and Nonrenewable Energy Consumption with Carbon Emission: A global study with the application of heterogeneous panel estimations. Renew. Energy 133, 685–691.

[25] Long X., Naminse E. Y., Du J., Zhuang J., 2015 Nonrenewable energy, renewable energy, carbon dioxide emissions and economic growth in China from 1952 to 2012. Renew. Sust. Energ. Rev. 52, 680–688.

[26] Yu S., Zheng S., Li X., 2018 The achievement of the carbon emissions peak in China: The role of energy consumption structure optimization. Energy Econ. 74, 693–707.

[27] Zhao X., Luo D., 2018 Forecasting fossil energy consumption structure toward low-carbon and sustainable economy in China: Evidence and policy responses. Energy Strateg. Rev. 22, 303–312.

[28] Muhammad B., 2019 Energy consumption, CO2 emissions and economic growth in developed, emerging and Middle East and North Africa countries. Energy 179, 232–245.

[29] Bilgili F., Koçak E., Bulut Ü., 2016 The dynamic impact of renewable energy consumption on CO2 emissions: A revisited Environmental Kuznets Curve approach. Renew. Sust. Energ. Rev. 54, 838–845.

[30] Dong K., Sun R., Hochman G., 2017 Do natural gas and renewable energy consumption lead to less CO2 emission? Empirical evidence from a panel of BRICS countries. Energy 141, 1466–1478.

[31] Pata U. K., 2018 Renewable energy consumption, urbanization, financial development, income and CO2 emissions in Turkey: Testing EKC hypothesis with structural breaks. J. Cleaner Prod. 187, 770–779.

[32] Nguyen K. H., Kakinaka M., 2019 Renewable energy consumption, carbon emissions, and development stages: Some evidence from panel cointegration analysis. Renew. Energy 132, 1049–1057.

[33] Shafiei S., Salim R. A., 2014 Non-renewable and renewable energy consumption and CO2 emissions in OECD countries: A comparative analysis. Energy Policy 66, 547–556.

[34] Boontome P., Therdyothin A., Chontanawat J., 2017 Investigating the causal relationship between non-renewable and renewable energy consumption, CO2 emissions and economic growth in Thailand. Energy Procedia 138, 925–930.

[35] Chen Y., Zhao J., Lai Z., Wang Z., Xia H., 2019 Exploring the effects of economic growth, and renewable and non-renewable energy consumption on China’s CO2 emissions: Evidence from a regional panel analysis. Renew. Energy 140, 341–353.

[36] Du Z., Lin B., 2018 Analysis of carbon emissions reduction of China’s metallurgical industry. J. Cleaner Prod. 176, 1177–1184.

[37] An R., Yu B., Li R., Wei Y., 2018 Potential of energy savings and CO2, emission reduction in China’s iron and steel industry. Appl. Energy 226, 862–880.

[38] Yang L., Lin B., 2016 Carbon dioxide-emission in China’s power industry: Evidence and policy implications. Renew. Sust. Energ. Rev. 60, 258–267.

[39] Du Q., Lu X., Li Y., Wu M., Bai L., Yu M., 2018 Carbon Emissions in China’s Construction Industry: Calculations, Factors and Regions. Int. J. Environ. Res. Public Health 15, 1220 10.3390/ijerph15061220 29890769 PMC6025463

[40] Xia F., Zhang X., Cai T., Wu S., Zhao D., 2020. Identification of key industries of industrial sector with energy-related CO2 emissions and analysis of their potential for energy conservation and emission reduction in Xinjiang, China. Sci. Total Environ. 708, 134587 10.1016/j.scitotenv.2019.134587 31787290

[41] Wang X., Duan Z., Wu L., Yang D., 2015 Estimation of carbon dioxide emission in highway construction: a case study in southwest region of China. J. Cleaner Prod. 103, 705–714.

[42] Tang C., Zhong L., Pin N., 2017 Factors that influence the tourism industry’s carbon emissions: a tourism area life cycle model perspective. Energy Policy 109, 704–718.

[43] Lin B., Lei X., 2015 Carbon emissions reduction in China's food industry. Energy Policy 86, 483–492.

[44] Feng C., Huang J., Wang M., 2018 The driving forces and potential mitigation of energy-related CO2 emissions in China's metal industry. Resour. Policy 59, 487–494.

[45] Du G., Sun C., Ouyang X., Zhang C., 2018 A decomposition analysis of energy-related CO2 emissions in Chinese six high-energy intensive industries. J. Cleaner Prod. 184, 1102–1112.

[46] Gao Z., Geng Y., Wu R., Chen W., Wu F., Tian X., 2019 Analysis of energy-related CO2 emissions in China's pharmaceutical industry and its driving forces. J. Cleaner Prod. 223, 94–108.

[47] Su B., Ang B. W., Li Y. 2017 Input-output and structural decomposition analysis of Singapore's carbon emissions. Energy Policy,105, 484–492.

[48] Liao M. Q., Xu L. M. 2017 IO-SDA Model of CO2 Emissions and its Empirical Research[J]. Statistical Research, 34, 62–70.

